# The Opioid Safety Toolkit: An interactive prescription opioid safety toolkit to increase opioid safety literacy and behaviours among people prescribed opioids for pain—a randomised controlled trial

**DOI:** 10.1111/add.70412

**Published:** 2026-04-14

**Authors:** Suzanne Nielsen, Frederick Fox, Tina Lam, Alex Waddell, Monica Jung, Bosco Rowland, Jessica Watterson, Dhruv Basur, Chris Prawira, Joshua Paolo Seguin, Patrick Olivier, Jarrod McMaugh, Paul Dietze, Louisa Picco

**Affiliations:** ^1^ Monash Addiction Research Centre (MARC), Eastern Health Clinical School Monash University Melbourne Victoria Australia; ^2^ Action Lab, Faculty of Information Technology Monash University Melbourne Victoria Australia; ^3^ School of Public Health and Social Development Deakin University Melbourne Victoria Australia; ^4^ Pharmaceutical Society of Australia Melbourne Victoria Australia; ^5^ Burnet Institute Melbourne Victoria Australia

**Keywords:** behaviour change, behavioural science, health literacy, implementation science, naloxone, opioid safety, RCT

## Abstract

**Background and aims:**

Prescription opioid‐related harm remains a significant public health concern. This study aimed to evaluate the efficacy of the Opioid Safety Toolkit, a co‐designed, interactive online resource, in increasing naloxone uptake and healthcare provider discussions among adults prescribed opioids for pain.

**Design:**

Parallel‐group, open‐label, randomised controlled trial.

**Setting:**

Community‐based, online recruitment across Australia.

**Participants:**

Adults (*n* = 314) prescribed opioids for non‐cancer pain.

**Interventions:**

Participants were randomised to receive either the Opioid Safety Toolkit (intervention, *n* = 152), which included interactive and tailored educational content on opioid safety, or an active control website presenting evidence‐based opioid safety information (*n* = 162). Both groups were followed for four weeks.

**Measurements:**

The primary outcome was self‐reported naloxone requests four weeks post‐intervention. Other outcomes were intentions to access naloxone immediately post‐intervention, and healthcare provider discussions about opioid safety at four weeks, opioid safety knowledge (immediately after the intervention and at four weeks), satisfaction with resources and naloxone possession at four weeks.

**Findings:**

Participants in the intervention group were more likely to have requested naloxone at four weeks compared with controls [21.7% vs 9.9%, odds ratio (OR) = 2.5, 95% confidence interval (CI) = 1.3, 4.8; *P* = 0.005], and more likely to report intentions to access naloxone immediately post‐intervention compared with controls (41.4% vs 15.4%, OR = 3.9, 95% CI = 2.3, 6.6; *P* < 0.001). Participants in the intervention group were not more likely to have healthcare provider discussions at four weeks compared with controls (OR = 1.1, 95% CI = 0.7, 1.8; *P* = 0.620). Post‐intervention opioid overdose knowledge was statistically significantly higher in the intervention group compared with control group (Mean score 16.6, 95% CI = 15.5, 17.7 vs control mean score 13.3, 95% CI = 12.3, 14.3). Satisfaction with the resource was higher in the intervention group compared with control group (Mean = 20.0, 95% CI = 18.7, 21.3 vs Mean = 18.0, 95% CI = 16.7, 19.3, *P* = 0.035).

**Conclusions:**

We found good evidence that, compared with a gold‐standard opioid information website, the Opioid Safety Toolkit increased naloxone requests among Australian adults prescribed opioids for non‐cancer pain. We also observed consistent effects across secondary outcomes, with the Toolkit increasing intentions to access naloxone, enhancing opioid overdose knowledge and yielding higher satisfaction ratings, although it did not increase healthcare provider discussions at four weeks.

## INTRODUCTION

Opioids are commonly used in most high‐income countries; a quarter of the US population used opioid analgesics in 2023 [1]. Over the past two decades, prescription opioid‐related harms in countries such as the USA, Canada and Australia have highlighted the need for interventions to increase their safer use [2, 3]. Naloxone is a medicine that safely and effectively reverses the effects of opioids when administered to a person who has overdosed. Studies have demonstrated that pharmacist‐led naloxone provision interventions can be effective in reducing opioid harms, such as overdose, as well as related opioid use risk behaviours [4, 5, 6].

However, a range of barriers to pharmacists discussing opioid risks and directly initiating naloxone provision have been identified [4, 7]. These include pharmacists’ concerns about patient perceptions of naloxone, pharmacists’ comfort talking about opioids and uncertainty about which patients to offer naloxone to [7]. An alternative to healthcare professional‐driven approaches is to develop interventions that directly target patient behaviours by increasing health literacy around opioid safety, encouraging them to understand the role of naloxone and increase their knowledge about safer prescription opioid use. People who are prescribed opioids often have limited understanding of opioid harms (and even low recognition of what an opioid is), yet when they are provided with clear explanations about interventions such as take‐home naloxone, there is high acceptance and interest [8]. Therefore, empowering people who are prescribed opioids for pain to directly request naloxone and ask for advice about their opioid use could overcome some of these barriers, while addressing established knowledge gaps among this population.

Existing, validated tools have been established to assist people prescribed opioids in identifying their own risks, and are both reliable and acceptable to consumers [4, 9, 10]. A pilot implementation study of opioid risk screening in community pharmacy settings demonstrated high acceptance and follow‐up rates for health advice [4]. This pharmacist‐led intervention, however, was reliant on pharmacists identifying patients and initiating screening, which occurred infrequently. A current gap exists in terms of how to effectively raise consumer knowledge and increase consumer‐driven behaviours that promote the safe use of opioids.

Furthermore, there is a need to better understand consumer preferences relating to the specific resources and formats that would aid and support increased health literacy and behaviour change. Currently, there is information available online that can be viewed passively, but studies using similar resources have found that passive access to health information has limited impact on behaviours related to safe opioid consumption [11]. In contrast, interventions that go beyond health literacy alone, such as interactive interventions discussing opioid risk, have had substantial effects on risk reduction [12].

Studies are needed to evaluate the efficacy of interventions targeted directly at consumers that are designed to empower their opioid safety behaviours. Whether more interactive resources could increase health literacy associated with opioid use is currently unknown. To address these gaps, we co‐designed an evidence‐based interactive Opioid Safety Toolkit with consumers and healthcare professionals that aims to: (i) have high acceptability among consumers; (ii) increase consumer health literacy regarding opioid safety; and (iii) increase opioid safety behaviours, such as accessing naloxone.

The Opioid Safety Toolkit was designed based on co‐design and implementation science approaches. This randomised controlled trial (RCT) sought to test the efficacy of the Opioid Safety Toolkit compared with an existing opioid information website (active control).

We hypothesised that, compared with controls, those randomised to the Opioid Safety Toolkit would report greater naloxone requests 4 weeks after the intervention (primary outcome).

## METHODS

### Design

This parallel‐group, open‐label, interventional RCT compared use of a co‐designed interactive toolkit, the Opioid Safety Toolkit, which included interactive and tailored educational content, with an existing consumer website, the National Prescribing Service (NPS) MedicineWise page on opioids and opioid safety. Exposure to the intervention or control condition was for approximately 30 minutes, with two follow‐up timepoints, the first immediately after the intervention, and the second at 4 weeks. The primary outcome was measured at 4 weeks. All study procedures were prospectively registered and all relevant methodological details for the trial are reported in the clinical trial registry (Clinical Trial Registration: ACTRN12624000176561) in place of a published protocol. This study has received ethics approval from Monash University Human Research Ethics Committee (ref. HREC 40988). The study findings are reported in accordance with the CONSORT (CONsolidated Standards of Reporting Trials) checklist for non‐pharmacological RCTs [13] (Table S1). The study was an open‐label study with no blinding to study condition.

### Participants

We recruited Australian adults aged at least 18 years who had been prescribed opioids for non‐cancer pain for at least 2 weeks and reported having taken opioids in the past 4 weeks. Participants were recruited via advertisements on social media and websites for the two relevant consumer pain groups in Australia (Painaustralia and Chronic Pain Australia), in addition to paid Facebook advertising. Social media advertising directed participants towards a study website, where people could leave their contact details to enable a researcher to send them a link to eligibility screening questions before providing them with access to a baseline survey, if eligible. Baseline surveys had extensive validity checks (e.g. ensuring logical combinations of responses and to identify duplicates), and self‐completed medication diaries were manually reviewed by qualified pharmacists to ensure that only eligible participants proceeded to randomisation.

Participants were entered into a prize draw to win one of two iPads at the completion of the baseline survey. Participants were offered an AUD$40 eGift card upon completing each of the follow‐up surveys (immediately after the intervention and at 4 weeks), as compensation for their time.

### Setting

Community‐based study in Australia, where naloxone is accessible for free through Australian community pharmacies to anyone who may be at risk of witnessing or experiencing an opioid overdose, through a nationally funded program, with broad national coverage [14].

### Procedures

Research Electronic Data Capture (REDCap) [15] was used for survey data capture and management. Participants completed three surveys: at baseline; immediately after the intervention; and at 4 weeks. Self‐administered survey instruments were used as these are known to maximise the disclosure of sensitive behaviours. Participants were also reassured that their responses were confidential. Baseline surveys were completed online, with responses from baseline surveys manually screened to confirm eligibility prior to randomisation. On randomisation, participants were emailed links to the relevant online resource (either the Opioid Safety Toolkit or the active control website). Participants were required to spend 30–60 minutes with the online condition that they were randomised to before completing two surveys, one immediately after viewing the resource and one 4 weeks later. Both groups were followed for 4 weeks. Follow‐up survey links were emailed to participants 1 hour after being provided with access to the randomised online resource, using contact details provided within the baseline survey. The final follow‐up survey (sent at 4 weeks) included a screenshot of the homepage of the online resource that participants viewed to assist participant recall about the study condition they had been randomised to. The timing that each of the measures was collected is outlined in Table S3. Baseline data collection began in June 2024, with the final week‐4 follow‐up survey submitted in August 2024.

### Randomisation

Eligible participants were randomised in a 1 : 1 ratio via a computer‐generated randomisation table using REDCap [15]. Participants were allocated to the intervention or control group by a member of the research team using the randomisation function in REDCap once a review of their baseline data confirmed their eligibility.

### Allocation concealment

Allocation was concealed using the REDCap randomisation module. An independent researcher, not otherwise involved in the trial, generated the randomisation schedule that was uploaded into REDCap. Study investigators had no access to the allocation sequence.

### Intervention

#### Toolkit (experimental condition)

The Toolkit design was guided by using the Theoretical Domains Framework (TDF) and using the Behaviour Change Techniques Taxonomy (BCTT) within the distinct phases of the Double Diamond design process [16]. The TDF is commonly used to understand the influences of behaviour, and comprises 14 domains (e.g. skills, social influences, beliefs about capabilities) that can be used in conjunction with the BCTT to design interventions to address behavioural influences [17]. Specifically, in the design of the intervention we used the TDF to identify barriers and facilitators to the safety behaviours associated with opioid use. The factors were then mapped to relevant BCTT items using the Theory and Techniques Tool [17, 18]. The use of the TDF and BCTT within a co‐design methodology ensured the intervention is theory‐ and evidence‐based as well as contextually appropriate [19]. The co‐design process involved consumers and professionals (consumer advocates, pain specialists, pharmacists, prescribers and researchers) across multiple workshops, followed by extensive user testing with consumers [16].

The resultant online interactive Opioid Safety Toolkit (saferopioiduse.com.au), includes: (i) developing a personalised Opioid Safety Plan (which includes interactive opioid risk information, education on the use of naloxone and how to access it, and prompts to share information about the plan and naloxone with people in the same home); and (ii) the Routine Opioid Outcome Monitoring (ROOM) tool [10], alongside additional pain management resources and general evidence‐based information on opioids. The website flow is designed so participants are initially directed to complete an Opioid Safety Plan and are then directed to complete the ROOM tool, with other key information sources on naloxone and opioids listed in a menu bar. As part of the Opioid Safety Toolkit participants were able to download or email to themselves a personalised Opioid Safety Plan, and their responses and feedback from completing the ROOM tool, to discuss with a healthcare professional later. Participants were informed it would take 30–60 minutes to review the material and complete the follow‐up survey, and they could return to the website at any time after their initial session. After randomisation, they received an email with a link to their allocated online resource and instructions to review the material before completing the follow‐up survey. Participants were unable to begin the follow‐up survey until they confirmed they had reviewed the material.

#### Control condition

The control condition was an existing website (NPS MedicineWise) with information on prescribed opioids and naloxone (https://www.nps.org.au/consumers/opioid-medicines) [20]. Participants were informed it would take 30–60 minutes to review the material and were asked to complete the follow‐up interview after initially reviewing the material, though they could return to the website anytime. As with the intervention, prior to completing the follow‐up survey participants were resent the link and asked to confirm they had reviewed it before commencing the survey.

### Measures

The outcomes reflect the aims of the Opioid Safety Toolkit; these were to empower consumers to increase their health literacy with respect to naloxone and opioid safety, and to support behaviour change.

#### Primary outcome

*Naloxone requested*: This was measured by asking whether the participant has requested naloxone (yes/no) within 4 weeks of the intervention.

#### Secondary outcomes

*Secondary outcome 1*: Intentions to access naloxone immediately after the intervention. This was measured with a dichotomous self‐report question about participant’s intention to access naloxone (yes/no).

*Secondary outcome 2*: Seeking information to support opioid safety from a healthcare provider. This was measured by asking whether participants had conversations with healthcare providers (e.g. pharmacists or doctors) about opioid safety (yes/no) at 4 weeks.

*Secondary outcome 3*: Naloxone and overdose knowledge immediately after the intervention. This was measured using 24 items from the Prescription Opioids Opioid Overdose Knowledge Scale (Rx‐OOKS), a validated instrument that measures knowledge about prescription opioid overdose and naloxone (α = 0.8495) [21]. The last two items on the instrument were not included in the Cronbach’s alpha calculation as they were measured on an incomparable scale.

*Secondary outcome 4*: Opioid risk behaviours. This was measured with three individual items, each of which measured a modifiable risk for prescription opioid overdose: (i) concurrent alcohol and opioid use; (ii) concurrent sedative and opioid pain medication use; and (iii) self‐escalating the amount of opioid pain medications used. Each item was measured on a five‐point scale, with response options of: never (0), rarely (1), sometimes (2), often (3) and very often (4).

*Secondary outcome 5*: Level of self‐assessed opioid risk. This was measured by the following question ‘How would you rate your level of risk of experiencing an opioid pain medication overdose?’ Responses were made on a four‐point scale of no risk (0), low risk (1), moderate risk (2) or high risk (3).

*Secondary outcome 6*: Time spent reviewing online resources. This was measured by self‐reported time spent reviewing online resources in minutes, reported immediately after the intervention, as a measure of adherence to the allocated intervention.

*Secondary outcome 7*: User satisfaction with the online resource was assessed with the modified version of the Questionnaire for Assessing User Satisfaction With Mobile Health Apps, which included minor wording adaptations to reflect the context of the intervention [22], using a seven‐item scale, with some measures reverse scored (Table S2). Scores are summed and range from a minimum of zero to a maximum of 28 (α = 0.8733).

*Secondary outcome 8*: Naloxone possession. This was measured by the participants indicating whether they had received naloxone after requesting it (yes/no). Where naloxone was not received, additional questions captured the reason for this.

*Secondary outcome 9*: Change in naloxone and overdose knowledge over time (assessed at all three timepoints) using Rx‐OOKs.

#### Other measures

*Demographics*: We collected participant demographic data on age, gender identity, housing, education, current work status and household income.

*Pain, pain history and opioid outcomes*: Data pertaining to the duration of pain and pain treatments (current and previous) were collected. The ROOM tool [10] was used to capture key opioid outcomes measures including pain, mood and opioid use disorder as follows. Current pain using PEG (a three‐item measure that captures pain severity, enjoyment and general activity) [23], a brief three‐item pain measure derived from the Brief Pain Inventory and validated in ambulatory care settings. Mood was measured using the Patient Health Questionnaire‐2 (PHQ‐2) [24]. Opioid use disorder was measured with OWLS, a four‐item opioid use disorder screening tool that measures: overuse (taking more than prescribed); worrying about use of opioid medicines; losing interest in usual activities owing to opioid medicines; and feeling slowed down, sluggish or sedated owing to opioid medicines in the past 3 months [9].

*Comorbidity questionnaire*: We used the self‐administered comorbidity questionnaire (SCQ) [25] to collect information on participant health conditions, as a validated way to gather information on self‐reported comorbidities when access to medical records is not feasible.

*Current medication use*: We collected information on current opioid use and converted the results into an oral morphine equivalent daily dose using an established approach [26]. We also collected information on participants’ duration of opioid use for chronic pain and documented concurrent use of other psychoactive medications, including tricyclic antidepressants, gabapentinoids, benzodiazepines, antipsychotics and tricyclic antidepressants.

*Substance use*: We measured alcohol and other substance use using the Alcohol, Smoking and Substance Involvement Screening Test (ASSIST) [27].

*Naloxone request*: Participants were asked if they had requested naloxone (primary outcome), and the outcome of their requesting naloxone (i.e. did they receive naloxone when requesting it). Participants who had not accessed naloxone at follow‐up were asked reasons for not accessing it.

*Changes to trial outcomes after the trial commenced*: On recommendation of the journal’s statistical editor, two outcomes initially specified as primary outcomes were re‐specified as secondary outcomes, to leave a single primary outcome. The trial registry was updated to note this change.

### Statistical analysis

Sample size was determined based on a test of difference between two independent proportions, with an estimated 20% of people in the intervention group meeting the criteria for the outcomes of: (i) intending to access naloxone immediately after the intervention; and (ii) requesting naloxone at 4 weeks, compared with 5% of the control condition. A sample size of at least 202 participants (101 per group) was determined to provide 90% power with an α of 0.05 (two‐tailed).

The analyses were conducted by an independent statistician who was blinded to the study conditions. The primary outcome was analysed using logistic regression comparing outcomes in the control and intervention groups 4 weeks after the intervention, following intention‐to‐treat (ITT) principles. For the primary outcome, missing data were coded as the worst outcome (e.g. did not request naloxone). This approach was selected as a conservative and plausible assumption in addiction research, where loss to follow‐up is often associated with poorer outcomes, and therefore reflects an assumption of missing not at random (MNAR).

Per‐protocol analyses were conducted as sensitivity analyses in a restricted sample, including only participants who did not have access to naloxone at baseline. These analyses were undertaken for the primary outcome, secondary outcome 1 (intention to access naloxone) and secondary outcome 8 (naloxone possession), to assess the robustness of findings among participants who could receive the intervention as intended. Where complete‐case analyses were undertaken, these rely on the contrasting assumption that data were missing completely at random (MCAR).

Secondary outcomes comparing a mean at a single timepoint were analysed using Student’s *t*‐tests. The same ITT approach was used for Student’s *t*‐tests performed at one timepoint as described for the primary outcome above. Linear mixed models were used where secondary outcomes were measured at multiple timepoints. This was done with a model that included the fixed categorical effects of assessment time, group indicator and treatment‐by‐group interaction. ITT for mixed models used maximum‐likelihood estimation, and assumed data were missing at random. For secondary outcomes 3–7 and 9, sensitivity analysis was undertaken using cases with no missing data (complete cases). All analyses were completed with Stata 18 (StataCorp LLC, College Station, TX, USA).

## RESULTS

We randomised 314 people to receive the Opioid Safety Toolkit intervention (*n* = 152) or the active control website (*n* = 162). Table 1 provides an overview of participant characteristics at baseline. Twenty‐eight participants in the control group (17.3%) and 30 participants in the intervention group (19.7%) reported having access to naloxone at baseline. Participants had a mean age of 49.1 years and 22.9% were male. The sample reported minimal non‐medical substance use (Table S3).

**TABLE 1:** Characteristics of sample by randomisation group at baseline.

	Control (*n* = 162)	Toolkit (*n* = 152)	Total (*n* = 314)
Mean age (SD)	49.1 (13.6)	49.0 (14.4)	49.1 (14.0)
**Gender, *n* (%)**			
Male	36 (22.2%)	36 (23.7%)	72 (22.9%)
Female^a^	126 (77.8%)	116 (76.3%)	242 (77.1%)
**Country of birth, *n* (%)**			
Australia	140 (86.4%)	132 (86.8%)	272 (86.6%)
Other	22 (13.6%)	20 (13.2%)	42 (13.4%)
**Jurisdiction living in, *n* (%)** ^b^			
Victoria	30 (19.2%)	36 (24.2%)	66 (21.6%)
New South Wales	41 (26.3%)	34 (22.8%)	75 (24.6%)
Queensland	38 (24.4%)	32 (21.5%)	70 (23%)
Western Australia	18 (11.1%)	18 (11.8%)	36 (11.5%)
South Australia	10 (6.2%)	19 (12.5%)	29 (9.2%)
Tasmania	12 (7.4%)	7 (4.6%)	19 (6.1)
Australian Capital Territory	6 (3.7%)	3 (2%)	9 (2.9%)
Northern Territory	7 (4.3%)	3 (2%)	10 (3.2%)
**Work status, *n* (%)**			
In paid work	52 (32.1%)	43 (28.3%)	95 (30.3%)
Unemployed	5 (3.1%)	6 (3.9%)	11 (3.5%)
Disabled/sick	55 (34%)	44 (29%)	99 (31.5%)
Completely retired	16 (9.9%)	20 (13.2%)	36 (11.5%)
Other	34 (21%)	39 (25.7%)	73 (23.2%)
**Median pain score (PEG) (IQR)**	7.0 (5.67, 8.00)	7.0 (6.00, 7.67)	7.0 (6.00, 7.67)
**Median years of opioid use (IQR)**	7.5 (4.2, 15.0)	7.3 (3.3, 15.0)	7.4 (4.0, 15.0)
**Median daily oral morphine equivalent (OME) dose (IQR)**	47.5 (21.0, 90.0)	52.5 (20.8, 120.0)	50.0 (21.0, 105.0)
**Current non‐opioid medications, *n* (%)**			
Benzodiazepines and Z‐drugs	51 (31.5%)	51 (33.6%)	102 (32.5%)
Gabapentinoids	41 (25.3%)	36 (23.7%)	77 (24.5%)
Other mental health medications	69 (42.6%)	89 (58.6%)	158 (50.3%)
Medical cannabis	25 (15.4%)	18 (11.8%)	43 (13.7%)
**Mean number of comorbidities (SD)**	4.3 (1.9)	3.6 (1.8)	4 (1.9)
**Positive screen for opioid use disorder, *n* (%)** ^c^	42 (25.9%)	48 (31.6%)	100 (31.8%)

^a^ Includes non‐binary participants (*n* = 6) and participants who preferred not to say (*n* = 2).

^b^ Sample size by state is broadly representative of state population.

^c^ Assessed using OWLS, a prescription opioid use disorder screening tool, with scores indicating that it is likely that symptoms of opioid use disorder are emerging and further assessment with a healthcare professional is warranted.

### Attrition

**FIGURE 1:**
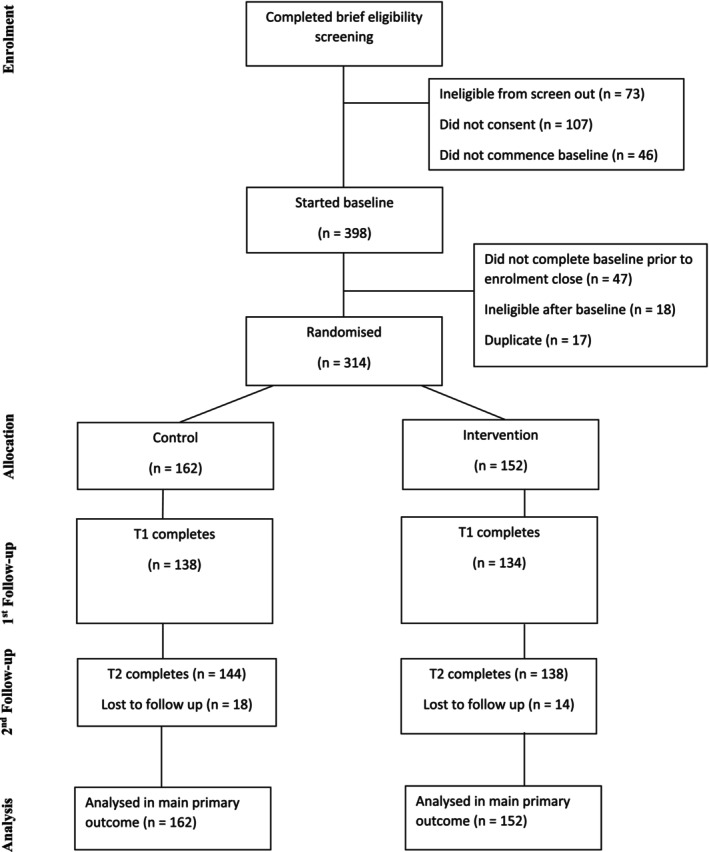
CONSORT (CONsolidated Standards of Reporting Trials) flow diagram.

Thirty three participants were lost to follow‐up at the final timepoint, with 15 participants lost to follow‐up in the intervention arm and 18 participants lost to follow‐up in the control group (Figure 1).

### Primary outcome

#### Requesting naloxone

The intervention group (*n* = 33/152) were more likely (OR = 2.5, 95% CI = 1.3, 4.8; *P* = 0.005) to report requesting naloxone at 4 weeks compared with those in the control group (*n* = 16/162) (Table 2).

**TABLE 2:** Intention‐to‐treat and per‐protocol analysis using proportions or means.

Outcomes	Intention‐to‐treat analysis (main analyses)			Sensitivity analysis (per‐protocol sample)		
	Intervention (*n* = 152)	Control (*n* = 162)	Between‐group difference	Intervention	Control	Between‐group difference
**Primary outcome**	**% (*n*)**	**% (*n*)**		**% (*n*)**	**% (*n*)**	
Requested naloxone (four weeks after intervention)	21.7 (33)	9.9 (16)	OR = 2.5 (95% CI = 1.3, 4.8) *P* = 0.005; *n* = 314	23.8 (29/122)	10.5 (14/134)	OR = 2.7 (95% CI = 1.3, 5.3) *P* = 0.005; *n* = 256^a^
**Secondary outcomes**						
Intention to access naloxone (immediately after the intervention)	41.4 (63)	15.4 (25)	OR = 3.9 (95% CI = 2.3, 6.6) *P* < 0.001; *n* = 314	51.6 (63/122)	18.7 (25/134)	OR = 4.7 (95% CI = 2.7, 8.1) *P* < 0.001; *n* = 256^a^
Healthcare provider discussions about opioid safety at 4 weeks	44.7 (68)	42.0 (68)	OR = 1.1 (95% CI = 0.7, 1.8) *P* = 0.620; *n* = 314	49.3 (68/138)	47.2 (68/144)	OR = 1.1 (95% CI = 0.7, 1.7) *P* = 0.730; *n* = 282

	M; SD; (*n*)	M; SD; (*n*)		M; SD; (*n*)	M; SD; (*n*)	
Naloxone overdose knowledge (immediately after intervention)	16.6; 6.8; (152)	13.3; 6.6; (162)	*t*(314) = 4.3; *P* < 0.001	18.8; 3.1; (134)	15.6; 3.8; (138)	*t*(270) = 7.52; *P* < 0.001
Time spent viewing the resource	25.6; 18.1; (152)	27.5; 20.8; (162)	*t*(314) = 0.85; *P* = 0.398	29.0; 16.4; (134)	32.2; 18.8; (138)	*t*(270) = −1.50; *P* = 0.136
Satisfaction with the resource	20.0; 8.3; (152)	18.0; 8.5; (162)	*t*(314) = −2.11; *P* = 0.035	22.7; 4.0; (134)	21.1; 4.4; (138)	*t*(270) = −3.08; *P* = 0.002

				Per‐protocol analysis		
	% (*n*)	% (*n*)		% (*n*)	% (*n*)	
Naloxone possession at 4 weeks	16.4 (24)	9.7 (15)	OR = 1.8 (95% CI = 0.9, 3.7) *P* = 0.083; *n* = 314	16.4 (20)	9.7 (13)	OR = 1.8 (95% CI = 0.9, 3.8) *P* = 0.114; *n* = 256^a^

^a^ Excluding those who previously had naloxone in their possession (*n* = 30, intervention; *n* = 28, control).

### Secondary outcomes

#### Secondary outcome 1: intention to access naloxone

The intervention group were more likely to report intending to access naloxone immediately after the intervention compared with the control group (41.4% vs 15.4%; OR = 3.9; 95% CI = 2.3, 6.6; *P* < 0.001).

#### Secondary outcome 2: seeking information to support opioid safety from a healthcare provider

The intervention group were not more likely to seek information to support opioid safety compared with the control group at 4 weeks (44.7% vs 42.0%; OR = 1.1; 95% CI = 0.7, 1.8; *P* = 0.620).

#### Secondary outcome 3: naloxone and overdose knowledge immediately after intervention

A Student’s *t*‐test adjusting for unequal variances between the groups indicated that there were significantly greater knowledge scores in the intervention group (mean = 16.6) compared with the control group (mean = 13.3) immediately after the intervention (Table 2).

#### Secondary outcome 4: opioid‐risk behaviours (concurrent use of depressant drugs and opioid dose escalation)

The group‐by‐time interaction in the linear mixed model indicated that the reduction in both groups did not significantly differ between baseline and 4 weeks (β = 0.03; *P* = 0.939; Table 3). A figure for the predicted marginal means for this mixed model is presented in Figure S1.

**TABLE 3:** Linear mixed models for opioid risk, self‐reported risk, and naloxone and overdose knowledge.

Outcome	β	P	95% CI	β	P	95% CI
	Intention‐to‐treat analysis (main analysis)			Sensitivity analysis		
**Opioid risk behaviour**						
Intervention	0.25	0.429	−0.37, 0.87	0.25	0.204	−0.14, 0.64
Time	0.46	0.095	−0.08, 1.01	−0.71	<0.001	−0.95, −0.46
Intervention–time	0.03	0.939	−0.75, 0.81	0.21	0.237	−0.14, 0.57
Constant	2.203	<0.001	1.77, 2.64	2.203	<0.001	1.94, 2.47
**Self‐reported risk**						
Intervention	0.04	0.635	−0.11, 0.18	0.035	0.635	−01.11, 0.180
Time 1	0.13	0.016	0.03, 0.24	0.13	0.016	0.023, 0.24
Time 2	−0.21	<0.001	−0.32, −0.11	−0.21	<0.001	−0.32, −0.11
Intervention–time						
Intervention–time 1	0.17	0.027	0.02, 0.33	0.17	0.027	0.12, 0.33
Intervention–time 2	0.18	0.021	0.03, 0.33	0.18	0.021	0.03, 0.33
Constant	0.62	<0.001	0.512, 0.72	0.62	<0.001	0.52, 0.72
**Naloxone knowledge**						
Intervention	−0.34	0.410	−1.13, 0.46	−0.34	0.410	−1.14, 0.46
Time 1	1.79	<0.001	1.28, 2.30	1.79	<0.001	1.28, 2.30
Time 2	1.98	<0.001	1.48, 2.49	1.98	<0.001	1.48, 2.49
Intervention–time						
Intervention–time 1	3.47	<0.001	2.74, 4.19	3.47	<0.001	2.74, 4.19
Intervention–time 2	2.43	<0.001	1.71, 3.14	2.43	<0.001	1.71, 3.14
Constant	13.76	<0.001	13.21, 14.32	13.76	<0.001	13.21, 14.32

#### Secondary outcome 5: self‐reported ratings of risk of opioid overdose

**FIGURE 2:**
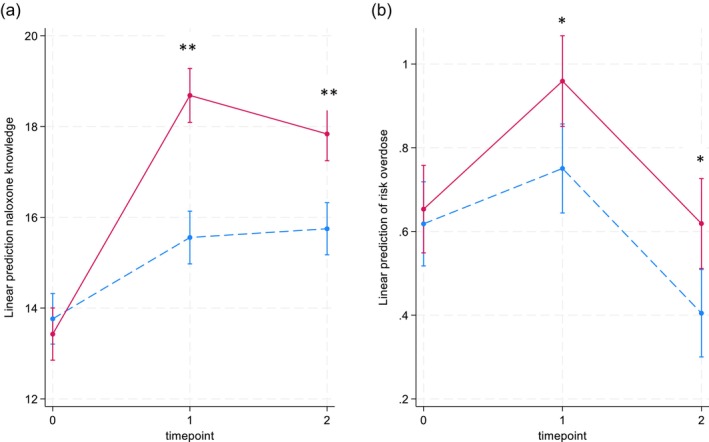
Self‐reported ratings of naloxone and overdose knowledge (a) and risk (b) at baseline, immediately after intervention and at 4 weeks. The *y*‐axis in panels (a) and (b) are predicted marginal means of mixed models for the secondary outcomes of naloxone knowledge and self‐reported risk, respectively; dashed blue lines = control group; solid red lines = intervention group; timepoint 0 = baseline; timepoint 1 = first follow‐up (immediately after the intervention); timepoint 2 = second follow‐up (at four weeks). The vertical lines on the intervention and control plots represent 95% confidence intervals. For both outcomes, there are significant differences (*P < 0.05; **P < 0.001, based on pairwise comparisons between groups at each timepoint).

Self‐reported ratings of risk changed significantly over time for both groups (Table 3). The group‐by‐time interaction indicated that there were significant differences between the two groups immediately after the intervention and at 4 weeks. Figure 2 presents the predicted marginal means of self‐reported ratings of risk from the linear mixed model. Pairwise contrasts indicate that self‐reported risks were significantly lower in the control group immediately after the intervention (by 0.21; *P* = 0.007) and at 4 weeks (by 0.21; *P* = 0.005). The adjusted difference in change from baseline (group‐by‐time interaction) indicated that the intervention group had a greater increase in self‐reported risk compared with the control group of 0.17 immediately post‐intervention (*P* = 0.027) and 0.18 at 4 weeks (*P* = 0.021).

#### Secondary outcome 6: time spent viewing the resource

Immediately after the intervention, the intervention group reported spending approximately 25.6 minutes (SD = 18.1 minutes) reviewing the online resource, while the control group reported spending 27.5 minutes (SD = 20.8 minutes). The difference in time spent viewing the resources between the groups was not significantly different.

#### Secondary outcome 7: satisfaction with the resource

Total satisfaction scores immediately after the intervention were significantly greater in the intervention group (mean = 20.0, SD = 8.3), compared with the control group (mean = 18.9. SD = 8.5).

#### Secondary outcome 8: naloxone possession

There was no significant difference in the number of participants who reported receipt of naloxone between the intervention group (16.4%) and the control group (9.7%) (Table 2). Participants who requested but did not receive naloxone were asked for the reason they did not receive it, with the most common reason being that naloxone was not being stocked where they had requested it.

#### Secondary outcome 9: naloxone and overdose knowledge over time

The group‐by‐time interaction indicated there was a significantly greater increase in knowledge over time (from baseline to 4 weeks) for the intervention group compared with the control group. Table 3 presents the results of the linear mixed model. A pairwise comparison indicated there was a significant difference of 3.13 (*P* < 0.001) immediately after the intervention and 2.08 (*p* < 0.001) at 4 weeks, between the intervention and the control groups. The adjusted difference in change from baseline (group‐by‐time interaction) was 3.47 (95% CI = 2.74, 4.19, *P* < 0.001) immediately post‐intervention and 2.43 (95% CI = 1.71, 3.14, *P* < 0.001) at 4 weeks. Figure 2 presents the predicted marginal means for naloxone and overdose knowledge for both groups from the linear mixed model.

##### Sensitivity analysis

A per‐protocol sensitivity analysis using only cases without missing data was undertaken with all primary and secondary outcomes. The model results were similar to the ITT analyses above (Tables 2 and 3), supporting the robustness and generalisability of the findings.

## DISCUSSION

We evaluated whether a low‐cost, evidence‐based and co‐designed interactive intervention, the Opioid Safety Toolkit, could increase behaviours related to opioid safety. We hypothesised that, compared with an active control group who were referred to a pre‐existing opioid safety website, those who were randomised to the toolkit would have increased self‐reported naloxone requests.

More than twice as many participants in the intervention group reported requesting naloxone compared with the control group, and almost three times as many participants in the intervention group reported an intention to obtain naloxone immediately post‐intervention. The toolkit design incorporated behaviour change techniques, such as the provision of personalised information on opioid safety, to enhance the uptake of opioid safety practices. This allowed participants to identify their own opioid‐related risks and develop individualised opioid safety plans, which often included keeping naloxone at home. These strategies appeared to enhance participants’ motivation and actions around opioid safety, as evidenced by the significant increase in reported naloxone requests. The use of the TDF and BCTT to design the intervention components ensured the toolkit content addressed multiple influences of safety behaviours [17, 18]. As such, the intervention includes theory‐ and evidence‐based components while being contextually relevant and appropriate for the needs of consumers [19].

The finding that the intervention group’s self‐assessed risk level increased following the intervention, compared with the control group, may suggest that co‐designed resources and individualised feedback fostered greater self‐awareness of opioid‐related risks, compared with accessing a static website with opioid information. The awareness of the participants’ own risks remained higher 4 weeks after the intervention. Our findings are consistent with prior research in emergency department settings, which found that personalised communication could reduce risk behaviours with opioids [12, 28]. The significantly higher satisfaction with the toolkit, compared with the control condition, further highlights the value of co‐designed interventions with consumer involvement, underpinned by evidence‐based behaviour change. Co‐designed interventions, which integrate consumer feedback during development, are increasingly recognised as effective in enhancing user engagement and outcomes [19].

Of note, despite clear evidence that the intervention drove increased requests for naloxone, the possession of naloxone was not significantly higher in the intervention group, possibly owing to systemic barriers, such as pharmacies not stocking naloxone. This finding highlights the need for targeted implementation efforts to improve pharmacy stocking of naloxone as opposed to a potential failure of the intervention itself. Although research has shown increases in the number of pharmacies stocking naloxone over time in Australia [14], a number of barriers to stocking naloxone in pharmacies have been identified [7]. Extensive efforts have been undertaken since trial completion to encourage pharmacies to stock naloxone to address these issues, alongside the promotion of the toolkit as a resource for consumers.

The hypothesis regarding healthcare provider discussions was not supported, with almost half of the participants in both groups reporting speaking to their healthcare providers within 4 weeks of either intervention. This result likely reflects the influence of the active control condition, which also provided opioid‐risk information and encouraged healthcare provider discussions.

Secondary outcomes demonstrated that the toolkit was more effective than the control condition in increasing knowledge about naloxone and opioid safety in the short to medium term. Future research should explore whether these effects persist over the long term or if ongoing interventions are required to maintain these improvements. A recent systematic review reported that providing knowledge about opioid overdose risk and naloxone can reduce risk and mortality in people who use illicit opioids [29], but there is limited work to show these same outcomes in people who are prescribed opioids. Given opioids are likely to remain a component of pain management for at least some patients, our study can make an important contribution in outlining effective interventions for increasing health literacy around overdose risk for this group.

As always, despite the use of a robust RCT design, there are limitations to consider. First, the reliance on self‐reported data may introduce recall or social desirability bias, despite efforts to assure participants of confidentiality. While self‐report data collection is often recommended in studies involving sensitive behaviours, it may affect the accuracy of some findings, though this is unlikely to differ between the groups. The careful screening on baseline surveys, inbuilt validity checks and confirmation of medication diary validity by a trained pharmacist prior to randomisation, strengthens our confidence in the data validity. Second, the lack of blinding in the intervention design could have influenced participant responses, though this was mitigated by using a robust active control condition. Third, the study population primarily consisted of participants recruited via social media and consumer organisations, potentially limiting the generalisability to individuals with different levels of health literacy or access to online resources. Fourth, while the study examined naloxone access, barriers such as pharmacy stocking were not directly addressed during the intervention, which may have affected outcomes related to naloxone possession. Finally, the relatively short follow‐up period precludes conclusions about the long‐term sustainability of the observed behaviour changes and knowledge improvements.

## CONCLUSION

We demonstrated that a co‐designed, interactive intervention significantly increased reported requests for naloxone among people prescribed opioids for pain, compared with an active control. The intervention also improved naloxone knowledge and increased intentions to access naloxone. The individualised feedback and behaviour change techniques of the intervention enhanced participant self‐awareness of opioid‐related risks. These findings highlight the potential of consumer‐targeted, interactive resources to complement healthcare provider‐driven initiatives in addressing prescription opioid safety. Future research should explore the long‐term efficacy of such interventions while also focusing on improving pharmacy engagement with naloxone supply.

## AUTHOR CONTRIBUTIONS

**Suzanne Nielsen:** Conceptualization; funding acquisition; methodology; project administration; supervision; writing—original draft; writing—review and editing. **Frederick Fox:** Data curation; formal analysis; investigation; validation; writing—original draft; writing—review and editing. **Tina Lam:** Methodology; project administration; supervision; writing—original draft; writing—review and editing. **Alex Waddell:** Methodology; resources; writing—review and editing. **Monica Jung:** Data curation; writing—review and editing. **Bosco Rowland:** Formal analysis; methodology; writing—review and editing. **Jessica Watterson:** Methodology; resources; writing—review and editing. **Dhruv Basur:** Methodology; software; writing—review and editing. **Chris Prawira:** Methodology; writing—review and editing. **Joshua Paolo Seguin:** Methodology; software; writing—review and editing. **Patrick Olivier:** Supervision; writing—review and editing. **Jarrod McMaugh:** Conceptualization; funding acquisition; writing—review and editing. **Paul Dietze:** Conceptualization; funding acquisition; methodology; writing—review and editing. **Louisa Picco:** Conceptualization; funding acquisition; methodology; writing—original draft; writing—review and editing.

## DECLARATION OF INTERESTS

All authors declare no competing interests.

## CLINICAL TRIAL REGISTRATION

ACTRN12624000176561.

## Supporting information

Box S1.
**Figure S1.** Intention‐to‐treat, predicted marginal means for opioid risk behaviour.
**Table S1.** CONSORT 2010 checklist of information to include when reporting a randomised trial.
**Table S2.** Modified version of the Questionnaire for Assessing User Satisfaction With Mobile Health Apps.
**Table S3.** Participant alcohol and other drug use as assessed using the ASSIST scale at baseline.

## Data Availability

The data that support the findings of this study are available on reasonable request from the corresponding author. The data are not publicly available due to privacy or ethical restrictions.
