# Supplementary material for: The Opioid Safety Toolkit: An interactive prescription opioid safety toolkit to increase opioid safety literacy and behaviours among people prescribed opioids for pain—a randomised controlled trial
Source: Addiction. 2026 Apr 14;121(8):2071–82. doi: 10.1111/add.70412 (PMC13357895; doi:10.1111/add.70412)
Supplement: Supplementary file 1 — Box S1. Figure S1. Intention‐to‐treat, predicted marginal means for opioid risk behaviour. Table S1. CONSORT 2010 checklist of information to include when reporting a randomised trial. Table S2. Modified version of the Questionnaire for Assessing User Satisfaction With Mobile Health Apps. Table S3. Participant alcohol and other drug use as assessed using the ASSIST scale at baseline. [file ADD-121-2071-s001.pdf]

Supplementary Material

The Opioid Safety Toolkit: an interactive prescription opioid safety toolkit to increase opioid safety literacy and behaviours among people prescribed opioids for pain – a randomised controlled trial.

Contents

Table S1: CONSORT 2010 checklist of information to include when reporting a randomised trial 2
Table S2. Modified version of the Questionnaire for Assessing User Satisfaction With Mobile Health Apps 4
Table S3. Timepoint for data collection domains 5
Table S4: Participant alcohol and other drug use as assessed using the ASSIST scale at baseline.................................. 6
Figure S1: Intention to Treat, Predicted Marginal Means for Opioid Risk Behaviour 7

Table S1: CONSORT 2010 checklist of information to include when reporting a randomised trial

Section/Topic	Item No	Checklist item	Reported on page No
**Title and abstract**			
	1a	Identification as a randomised trial in the title	Page 1
	1b	Structured summary of trial design, methods, results, and conclusions (for specific guidance see CONSORT for abstracts)	Page 3
**Introduction**			
Background and objectives	2a	Scientific background and explanation of rationale	Page 4
	2b	Specific objectives or hypotheses	Page 4
**Methods**			
Trial design	3a	Description of trial design (such as parallel, factorial) including allocation ratio	Page 5 & 6
	3b	Important changes to methods after trial commencement (such as eligibility criteria), with reasons	N/A
Participants	4a	Eligibility criteria for participants	Page 5
	4b	Settings and locations where the data were collected	Page 4
Interventions	5	The interventions for each group with sufficient details to allow replication, including how and when they were actually administered	Page 6 & 7
Outcomes	6a	Completely defined pre-specified primary and secondary outcome measures, including how and when they were assessed	Page 6 & 7
	6b	Any changes to trial outcomes after the trial commenced, with reasons	Page 8
Sample size	7a	How sample size was determined	Page 9
	7b	When applicable, explanation of any interim analyses and stopping guidelines	N/A
Randomisation:			
Sequence generation	8a	Method used to generate the random allocation sequence	Page 5
	8b	Type of randomisation; details of any restriction (such as blocking and block size)	Page 5
Allocation concealment mechanism	9	Mechanism used to implement the random allocation sequence (such as sequentially numbered containers), describing any steps taken to conceal the sequence until interventions were assigned	Page 5
Implementation	10	Who generated the random allocation sequence, who enrolled participants, and who assigned participants to interventions	Page 5-6
Blinding	11a	If done, who was blinded after assignment to interventions (for example, participants, care providers, those assessing outcomes) and how	Page 8

	11b	If relevant, description of the similarity of interventions	N/A
Statistical methods	12a	Statistical methods used to compare groups for primary and secondary outcomes	Page 8 & 9
	12b	Methods for additional analyses, such as subgroup analyses and adjusted analyses	Page 9, Supplementary material Box 1
**Results**			
Participant flow (a diagram is strongly recommended)	13a	For each group, the numbers of participants who were randomly assigned, received intended treatment, and were analysed for the primary outcome	Page 9
	13b	For each group, losses and exclusions after randomisation, together with reasons	Page 6, 7 & 18
Recruitment	14a	Dates defining the periods of recruitment and follow-up	Page 6
	14b	Why the trial ended or was stopped	N/A
Baseline data	15	A table showing baseline demographic and clinical characteristics for each group	Page 9 & 10
Numbers analysed	16	For each group, number of participants (denominator) included in each analysis and whether the analysis was by original assigned groups	Page 10
Outcomes and estimation	17a	For each primary and secondary outcome, results for each group, and the estimated effect size and its precision (such as 95% confidence interval)	Page 10, 11 & 12
	17b	For binary outcomes, presentation of both absolute and relative effect sizes is recommended	N/A
Ancillary analyses	18	Results of any other analyses performed, including subgroup analyses and adjusted analyses, distinguishing pre-specified from exploratory	N/A
Harms	19	All important harms or unintended effects in each group (for specific guidance see CONSORT for harms)	N/A – No harms were reported
**Discussion**			
Limitations	20	Trial limitations, addressing sources of potential bias, imprecision, and, if relevant, multiplicity of analyses	Page 15
Generalisability	21	Generalisability (external validity, applicability) of the trial findings	Page 15
Interpretation	22	Interpretation consistent with results, balancing benefits and harms, and considering other relevant evidence	Page 13 & 14
**Other information**			
Registration	23	Registration number and name of trial registry	Page 3 & 5
Protocol	24	Where the full trial protocol can be accessed, if available	Page 4
Funding	25	Sources of funding and other support (such as supply of drugs), role of funders	Page 1

Table S2. Modified version of the Questionnaire for Assessing User Satisfaction With Mobile Health Apps

	Strongly Disagree *1*	*2*	*3*	*4*	***Strongly Agree*** *5*
**What did you think about using the online resource?**					
It was easy to use					
It was good to use					
The time spent using it has been acceptable					
The introduction of how to use it was sufficient					
It was too time consuming					
It was boring to use					
I would recommend it to others					

Table S3. Timepoint for data collection domains

	Baseline	Immediately after intervention	Four weeks after intervention
Demographics	X		
**Main primary outcome**			
Requested naloxone			X[#]
**Secondary outcomes**			
Intentions to access naloxone	X	X	X
Healthcare provider contact about opioids or pain management	X		X
S1 & S7: Naloxone and overdose knowledge (Prescription Opioids Opioid Overdose Knowledge Scale [Rx-OOKS])	X	X	X
Overdose Risk Behaviours	X		X
Self-assessed overdose risk level	X	X	X
Time spent viewing resources		X	
Satisfaction with online resource		X	
Naloxone possession	X		X
**Other measures**			
Pain and pain history	X		
Self-administered co-morbidity questionnaire (SCQ)	X		
Current medication use	X		X
Routine Opioid Outcome Monitoring (ROOM) Tool	X		X
Substance use: Alcohol, Smoking and Substance Involvement Screening Test (ASSIST)	X		X

Supplementary material

Table S4: Participant alcohol and other drug use as assessed using the ASSIST scale at baseline

	Control (n = 162)	Intervention (n = 152)	Total (n=314)
In the past month, did you smoke a cigarette containing tobacco?	47 (29.0%)	38 (25.0%)	85 (27.1%)
Did you usually smoke more than 10 cigarettes each day?	25 (15.4%)	23 (15.1%)	48 (15.3%)
Did you usually smoke within 30 minutes after waking?	32 (19.8%)	26 (17.1%)	58 (18.5%)
In the past month, did you have a drink containing alcohol?	66 (40.7%)	67 (44.1%)	133 (42.4%)
On any occasion did you drink more than 4 standard drinks of alcohol?	21 (13.0%)	18 (11.8%)	39 (12.4%)
Have you tried and failed to control, cut down and stop drinking?	7 (4.3%)	7 (4.6%)	14 (4.5%)
Has anyone expressed any concern about your drinking?	6 (3.7%)	2 (1.3%)	8 (2.5%)
In the past month, did you use cannabis?	34 (21.0%)	30 (19.7%)	64 (20.4%)
Have you a strong desire or urge to use cannabis at least once a week or more often?	15 (9.3%)	14 (9.2%)	29 (9.2%)
Has anyone expressed concern about your use of cannabis?	3 (1.9%)	4 (2.6%)	7 (2.2%)
In the past month, did you use an amphetamine-type stimulant, or cocaine, or a stimulant medication not as prescribed?	2 (1.2%)	4 (2.6%)	6 (1.9%)
Did you use a stimulant at least once each week or more often?	0 (0%)	2 (1.3%)	2 (0.64%)
Has anyone expressed concern about your use of a stimulant?	0 (0%)	1 (0.7%)	1 (0.3%)
In the past month, did you use a sedative or sleeping medication not as prescribed?	11 (6.8%)	7 (4.6%)	18 (5.7%)
Have you had a strong desire or urge to use a sedative or sleeping medication at least once a week or more often?	4 (2.5%)	4 (2.6%)	8 (2.5%)
Has anyone expressed concern about your use of a sedative or sleeping medication?	1 (0.62%)	2 (1.3%)	3 (0.96%)
In the past month, did you use a street opioid (e.g. heroin) or an opioid-containing medication not as prescribed?	3 (1.9%)	3 (2%)	6 (1.9%)
Have you tried and failed to control, cut down or stop using an opioid?	0 (0%)	0 (0%)	0 (0%)
Has anyone expressed concern about your use of an opioid?	1 (0.62%)	2 (1.3%)	3 (0.96%)
Did you use any other psychoactive substance?#	4 (2.5%)	5 (3.3%)	9 (2.9%)

#Responses included mushrooms (n = 2), mirtazapine, caffeine, psilocybin, prescribed psychostimulants sertraline and sodium valproate

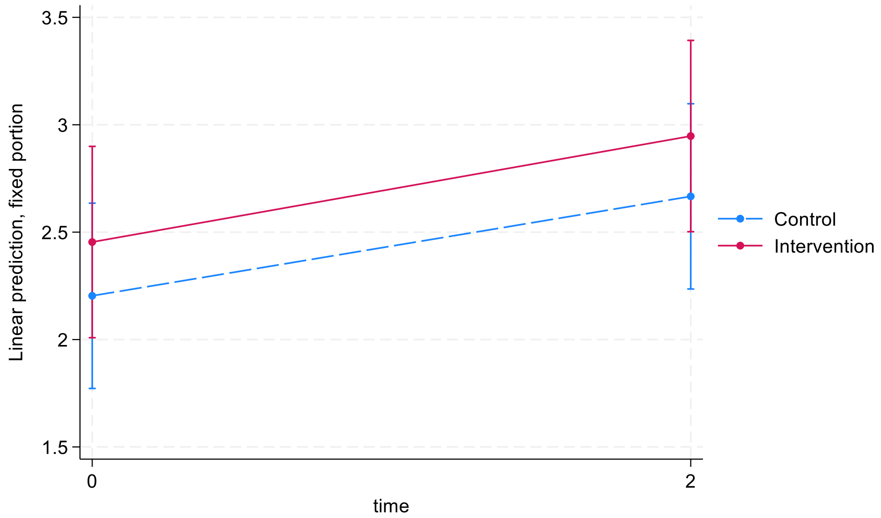

Notes: Y-axis is predicted marginal means of mixed models for predicted opioid self-reported risk. Timepoint 0 = baseline; time point 2 = second follow up. Vertical lines on interventon and control plots represent 95 confidence intervals. There are no signficant differences between intervention and control at time point 2, compared to baseline.

Figure S1: Intention to Treat, Predicted Marginal Means for Opioid Risk Behaviour
